# Laughter and effective presidential leadership: A case study of Ronald Reagan as the ‘great communicator’

**DOI:** 10.1371/journal.pone.0301324

**Published:** 2024-04-17

**Authors:** Patrick A. Stewart, Reagan G. Dye, Carl Senior

**Affiliations:** 1 Department of Political Science, University of Arkansas, Fayetteville, AR, United States of America; 2 Department of Political Science, George Washington University, Washington, DC, United States of America; 3 School of Psychology, Aston University, Birmingham, United Kingdom; National Taiwan University, TAIWAN

## Abstract

Former United States President Ronald Reagan’s use of media and his charismatic connection with viewers earned him the moniker “the great communicator”. One aspect of his charisma, the influence of elicited laughter, during a highly critical 5-minute news story by CBS reporter Leslie Stahl during the 1984 US presidential election is examined here. Two experiments examining the effects of audience laughter on perceptions of charismatic leadership are reported. In the first experiment the effects of audience laughter in response to Reagan’s comments were investigated. Here, Reagan’s perceived warmth as an effective leader significantly diminished when strong laughter is removed, whereas perceptions of competence remained unaffected. The second study carried out on an older cohort replicated and extended the first in a pre-registered design by considering the perception of trait charisma. Here, the presence or absence of audience laughter did not affect judgements of charisma. Additionally, the affective response before, and then after, the presentation of the news story was measured. Emotions associated with a positive appraisal all decreased after being shown the news story while emotions associated negative appraisal all increased. However, only participant anger was significantly increased when audience laughter was removed. Taken together the findings of both studies converge on the fact that subtle changes in media presentation of political leaders can have a significant effect on viewers. The findings show that even after 40 years in office the social psychological effects of presidential charisma can still influence observers.

## Introduction

One of the most important areas of study regarding politics and social psychology considers how social behaviours affect political interaction and, more specifically, how nonverbal signals influence perceptions of political leaders, especially as presented in television news. With the introduction of high-definition portrayals and ubiquitous hand-held devices, the role of the visual media in the portrayal of political leaders has grown. Experimental research on the visual primacy effect has also demonstrated that when there is conflicting information between the verbal and nonverbal channels in an audio-visual presentation, viewers have difficulty processing the verbal attributes of television news reports and remember the visuals with far more fidelity [1]. More attention is also paid to affectively important nonverbal communication when the nonverbal attributes of televised leader displays appear inappropriately matched to the rhetorical context [2–4].

The majority of research concerning the influence of nonverbal leader communication on social perception focuses on how visual attributes of posture, body movements, and facial display behaviours affect viewer perceptions and trait attributions [5]. At the same time, there is a small but growing literature that considers the role played by audible signals. Specifically, research considering the influence of the observable audience response to political figures suggests there is a significant intra-audience effect of emotional and evaluative signalling on other audience members while watching mediated events [6]. Applause-cheering, laughter, booing, chanting and combinations of these audible signals significantly influence how audience members view the televised political event or news coverage [6]. Viewers may unknowingly monitor and respond to the expressed intensity and type of follower utterance in support or opposition to the speaker and their stated political positions [7]. Media audiences, whether streaming debates, watching on television, or viewing through other media platforms, and perhaps more crucially, journalists who may be reporting on the event, may likewise be influenced by information conveyed via this audible channel. In other words, the social influence asserted through a specific audience response is similar to emotional contagion effects [6] and can affect viewer and listener perceptions with or without express awareness. However, the specific influence of different audience responses such as applause-cheering, laughter, and booing has yet to be studied in depth.

Despite the influence that observable audience responses may have on perceptions of leaders, systematic evaluation of these behaviours to political figures and how they affect the efficacy of politician narratives is limited. The few studies providing insight into the social influence of audience behaviour on political figures and policy issues tend to incorporate both audible and visually observable responses. Wiegman’s field experiment involved a videotaped studio audience either reacting positively, negatively, or neutrally to a well-known Dutch politician through audible utterances and different visible nonverbal behaviours [8]. Likewise, Fein and colleagues’ experiments considering Ronald Reagan’s second 1984 presidential debate performance did not differentiate between applause and laughter nor the moderator’s verbal and nonverbal response [9]. A study by Axsom and colleagues considering the verbal channel alone with regards to specific policy issues (e.g., imprisonment/probation) provided for comparison of “enthusiastic applause-cheering” to unenthusiastic and polite applause with occasional derisive cries [10] and found a tendency towards a simple consensus heuristic to make social judgments. Thus, while the limited research regarding political candidates and issues provides useful insights, it does not differentiate between different observable audience response types and often conflates visual and audible stimuli. In the work that follows we focus on one form of observable audience response–laughter–considering first its evolutionary roots and social influence briefly before focusing on its presence in politics and the types of humour that might elicit this type of behavioural response. We then evaluate group laughter’s role in providing a heuristic by which individuals may evaluate a political figure in ambiguous situations.

### Laughter

Laughter has been studied extensively across a broad range of social contexts with a wide range of approaches and techniques. Indeed, it is one of the few positive emotions considered in great detail, likely due to the social and survival benefits it confers. Across such species as canines [11], rats [12], and multiple primate species [13]–including humans–laughter signals playfulness, and with it benign intent [14]. In other words, social animals are more likely to cooperate and learn when in a playful state of mind as signalled by laughter.

Within humans, laughter emerges early and is seen across different cultures. Spontaneous laughter is observed in infants as young as 17 to 26 days [15], well before socially stimulated laughter occurs at three-to-four months of age [16]. Laughter is also observed within individuals who are blind since birth suggesting a possible adaptive function in social bonding [17].

The study of laughter is thus rightfully situated as a social phenomenon and would benefit from application of multiple different types of inquiry techniques; despite this, the primary method for analysing laughter has been naturalistic observational studies. Here, the effects of laughter tend to be studied in its social ecology. For instance, the ground-breaking work of Provine and colleagues took a “side-walk scientist” approach to laughter, finding that its role as a social lubricant by which mutual conversational grooming occurred was underappreciated, whereas its role as response to humorous comments was over-stated [16].

Laughter can be seen as socially influential due in large part to it being reliably identified through audible and visual characteristics. When nonverbal signals are easily and accurately identified, the more they are likely to affect perceptions and behaviour by being part of a highly learned (near automatic) repertoire of behaviours and responses that are likely to have been evolutionarily selected for survival purposes [18]. Thus, accurate recognition of the emotional state and behavioural intent of communicators provides relevant social information that influences perceptions and evaluations of others [19].

Reliable indicators of emotion may be defined as first, leading to an accurate recognition of the emotional state of the communicator, along with their resultant behavioural intent (e.g., bonding), and second, providing an index of the sender’s underlying state as one that is costly to produce [20]. Such signals are emotionally costly to produce due to their communicating underlying physiological states potentiating specific behaviour; furthermore, even when such signals are faked, physiological change can and does occur through the posing or acting out of these display behaviours.

Laughter may be classified as a costly, and hence reliable, signal when evoked or when it is difficult to control; even when faked, the initially emitted laughter leads to physiological change [21]. Individual laughter likewise serves as a social emollient by affecting perceiver mood states by dampening negative affect, increasing positive affect (and pain tolerance), while increasing social cooperation and group identity [22]. Laughter thus serves as a highly reliable social signal regarding behavioural intent [14].

### Laughter across differing social contexts

Because laughter provides a mechanism for the facilitation of affiliative social interactions that go beyond physical contact and is inclusive of large numbers of individuals, it should be easily and accurately recognized to indicate the underlying behavioural intent of the senders. Socially important utterances, such as laughter, can be seen as stereotyped activities by having coherent and identifiable vocalic, facial and even postural displays reliably associated with them. As pointed out by Gaspar and colleagues, the multimodal nature of this affiliative display behaviour, together with its early emergence in ontogenetic development and its stability throughout an individual’s lifespan, make it a predictable and reliable signal even as context changes [13]. Thus, due to the important role it plays in facilitating extended social interaction laughter may be one of the most reliable of nonverbal signals [23].

That is not to say that laughter cannot function in varying contexts, or convey differing or nuanced information, rather, that it is reliably recognized across cultures. Research regarding laughter at the individual level focuses on the role of such expressiveness being a pervasive social signal during interpersonal interactions. Here, laughter may serve to punctuate speech and indicate turn-taking and transitions within conversations [24, 25].

Laughter by individuals indicates social intent through the conveyance of vocalic qualities. Voiced laughter, with its sing-song characteristics, can communicate the experience of amusement, contempt, and even schadenfreude [26, 27]. Unvoiced laughter on the other hand, with its gruntlike characteristics [28], can be seen as signalling more competitive intent by being connected with aggressive statements [29]. This is perhaps due to the interrelationship between vocalic and facial movements seen with laughter and the amusement smile; facial display behaviour immediately after laughter-eliciting comments help convey social intent by punctuating the preceding statement [30]. In summary, laughter at the individual level serves a multitude of social functions based upon reliable multi-modal nonverbal signalling that is easily recognized. At the same time, the nuanced expression of individual laughter allows for subtle differences in to be conveyed in its meaning.

### Intra-audience effects of audience laughter

Laughter, as an important communicative signal, should also be socially contagious, or at least mimicked, to allow for the cohesion, broadening, and building of groups. Hatfield and colleagues [31] define social contagion as the “tendency to automatically mimic and synchronize facial expressions, vocalizations, postures, and movements with those of another person’s and, consequently, to converge emotionally” (p. 169). Thus, laughter would meet the definition of a socially contagious behaviour and indeed might provide the modal behaviour by meeting each of the above criteria in the convergence of mimicry and emotional response [13]. While group laughter is readily identifiable and distinct from other types of audience responses, it does not appear to have distinguishable characteristics that allow for the differentiation of members of different social groups from each other [32], nor in identifying nuanced social intent, as is the case with individual laughter.

Experimental research considering how individuals respond to group laughter tends to focus mainly on perceptions of how funny a stimulus is, whether visually with cartoons and written jokes [33–37], audio tapes of jokes, funny stories and stand-up routines [38–46], bloopers [47, 48], or scenes from television shows and movies [49–52]. When the source of the humour is taken into account, findings show that group laughter leads to individuals within the group being perceived more favourably across multiple dimensions relevant to leadership, including potential for success [45], authoritativeness, character, dynamism and interestingness [41], and credibility, likability, and lowered aggressiveness [53].

While each of the above studies were influenced by multiple factors, Vraga and colleagues [53] incisively comment that, “a humorous cue might be more important when faced with a more ambiguous context… as people have substantially less information on which to rely” (p. 145). Much of this research focuses on entertainment figures in which preconceptions either do not play a role due to low awareness, or by being so heterogeneous as to be randomly distributed. Political figures are different. Not only does their humour play a role in audience response, their group membership and social status predisposes perceptions [45]. Politicians, through their leadership role in society, belong to a clearly demarcated social group that is defined by a more restrictive set of social rules. Thus, the effects of receiving and perceiving laughter within a political context could be manifestly greater than in a non-political context, where it is expected and therefore part of the routine dialogue.

### Political laughter

When one considers group level behaviour in political contexts, research regarding observable audience responses tends to focus on the target and intent of verbal statements rather than the social influence process that laughter facilitates [30, 54, 55]. Current analyses describe audience response to political figures, by considering the length, strength, and intensity of audience laughter during political events [6, 56, 57]; however, the results reflect descriptive and correlational findings regarding response to individual speakers rather than group-related outcomes.

In group interactions, laughter is arguably more stereotyped and easily identified than other types of observable audience responses. The vocalic utterances that constitute laughter are much shorter in duration than applause-cheering, for instance. Analysis shows that group laughter in political contexts lasts on average 1–3 seconds in comparison with 2–8 seconds for applause-cheering [56–59]. Booing, another form of observable audience response, is surprisingly rare in political contexts. Interestingly, when an audience shows their appreciation for a humourous comment, applause-cheering prolongs the laughing response [30, 59]. This points to high levels of social mimicry in the case of group laughter, and then likely social contagiousness through its continuation via applause.

Studies regarding the use of humour during US presidential primary debates in 2008 [30] and the 2016 general election presidential debates [6, 56, 57] suggest that the main targets of humour during electoral campaigns tend to be out-group members. Here, ridicule and other forms of disparagement humour are used as a form of political rhetoric. In addition, self-deprecatory humour, where speakers poke fun at themselves or other in-group members, also occurs with regularity. The use of these different types of humour, ridicule and self-deprecation, likely holds strategic value, as ridicule can be used to derogate the competition or set normative boundaries on behaviour. On the other hand, self-deprecatory humour is useful for making a candidate more likable [60].

While there is an emerging body of research examining the type of humour employed by political candidates and the strength and duration of the laughter response, the correlational nature of this work limits the kinds of inferences that may be drawn. Furthermore, failed humour–which may be defined by the absence of laughter, its muted presence, or even booing–is rarely studied due to the difficulty of identifying enough occurrences for analysis [61].

The experimental research discussed in the previous section suggests that audience laughter certainly affects perceptions of humorousness and trait evaluations of the speaker. A number of scholars have shown that audience responses affect perceptions of political candidates [6, 8, 9]; however, the question remains as to how robust a role laughter, and the eliciting humour, plays in perceptions of political figures.

This question may be elaborated by considering what leadership traits are influenced by group laughter—and in what direction. The perception of competence and warmth are considered central to the identification and choice of leaders [62–66]. At the same time, these traits may be moderated or mediated by perceptions of leader charisma [67–72]. In the present study the effects of the observable audience response of laughter on the perception of trait charisma is examined by considering an individual considered to be amongst the most charismatic of presidents in United States history, Ronald Reagan.

### Humour types and political laughter

When humour in conjunction with laughter has been experimentally studied, the stimuli has tended to have been presented to the participants in the written form [73]. In other words, vignette studies varying the type of humour used, in combination with the asserted presence of laughter (or its absence), indicates success or lack thereof. As observed by Bitterly, Brooks, and Schweitzer in their extensive analysis of the effect of humour on interpersonal status [73]:

*Though humor can boost status*, *using humor is risky*. *Humor attempts can fail in several ways*: *by being too boring (i*.*e*., *not funny)*, *too bold (i*.*e*., *inappropriate)*, *or failing to elicit laughter from the audience*. *How the audience reacts profoundly influences perceptions*. *If the audience does not laugh*, *observers are less likely to view the humor attempt as appropriate or funny*, *and the joke teller may lose status*. (p. 17)

While the work of Bitterly and colleagues’ is indeed informative, their use of written vignettes as experimental treatments limits generalizability. Likewise, their focus on inappropriate humour relied upon sexually-charged quips; while important for the workplace with mixed sexes and fluid power dynamics, this type of humour is not used much by politicians in our technologically mediated era [30, 74]. Indeed, the use of sexualized humour in today’s political climate would probably be unsuccessful in eliciting laughter but would also likely alienate a substantial proportion of the electorate.

Regardless, their focus on perceptions of competence and status in response to humour–and the laughter that it elicits–is applicable to contests for leadership within politics. This is especially the case in viewer observations regarding leader competence, which in addition to perceptions of prestige is key to understanding why followers defer to, and confer status on, potential leaders.

Existing research in the use of humour by political figures suggest that it is used to either attack opponents, often through ridicule, or make light of oneself or allies [30, 56, 75, 76]. Smith and Powell found in the case of other- and self-disparaging humour by group leaders that those making ridicule attempts directed downwards at lower status group members were perceived as less effective, less encouraging, less helpful, and less socially attractive than those using self-directed humour [77]. However, this investigation also showed that not using humour was perceived as leading to better outcomes; in almost all leadership-based attributions that were considered save for tension relief and opinion offering, leaders who did not attempt any humorous remarks were perceived in a more positive light.

Arguably, the key factor here is the presence or absence of the laughter that is recognized to be an observable audience response to the politician. In the case of other-deprecatory humour, ridicule may increase perceived competence by virtue of martialling an audience together in their response to a tangible target; likewise, failure would see its reduction, negatively affecting the joke-teller. On the other hand, self-deprecatory humour successfully eliciting audience laughter would presumably lead to greater perceptions of warmth and communication effectiveness for the joke-teller [77]. Ultimately, observable (here audible) support for specific leader comments helps followers to identify leadership potential and other related traits.

### Ronald Reagan’s leadership style

Former US President Ronald Reagan’s moniker as “The Great Communicator” inspired a large body of literature assessing his communication style and its effects on public perceptions and the expectations of the American presidency [78]. As the first “celebrity” politician, Reagan provides insight into the role of media notoriety in politics. Consequently, re-examining Reagan’s relationship with the press and his ability to manipulate public perception is relevant in the current American political climate. The return of the celebrity presidency with the ascension of Donald Trump further warrants an historical examination of Reagan to glean insight into his unique communication style and public perception of populist leaders.

Reagan’s leadership style developed from his natural ability to connect with audiences and years of experience as a recognized film actor and television personality [79]. Upon entering national politics, Reagan was successful in enjoining his conservative agenda with the Republican Party establishment, garnering successful victories in the 1980 and 1984 presidential elections, and passing supply side economic policies. Although he suffered from periods of public scrutiny during his time in office, he was known as the “Teflon president” for his ability to rebound from criticism and controversy and gainfully employed rhetorical strategies to develop a reputation as humorous, charismatic, and likeable [75]. Now some 30 years since the Reagan era, the study of Reagan’s communication and leadership style has much to offer our current understanding of the normative behaviour of presidents and candidates operating under conditions of constant media scrutiny. Whereas Reagan was adept at connecting with Americans through television, contemporary office holders (and presidential hopefuls) must be able to compete with the flood of media choices now available across numerous platforms [80] and the fast pace of the issue-attention cycle.

Now more than ever, Converse’s assertion that the public pays more attention to, and takes cues from individuals in politics, rather than to politics and policy making itself is apparent in the individual-centred nature of the contemporary political environment [81]. If politicians possess the capacity to control the political agenda and how they are perceived by voters, then they have the ability to “go public” without relying on the mass media to set the agenda [82]. The specific case of Leslie Stahl’s mini-documentary on Reagan from the 1984 campaign is exemplary of this ability to skirt around the media narrative and control perceptions simply through imagery and audience response. Reagan’s mastery of image management in relation to television, including the use of self-deprecatory humour and direct appeals to supporters, provides a blueprint for understanding how presidential contenders must operate to maximize effectiveness in today’s hybrid media era [83].

The case study approach employed provides a historically relevant example that is recognized by many political communication scholars as a turning point in how nonverbal behaviour and social signals are considered [84, 85], it also presents an emotionally evocative stimuli that better reflects the “real world” of media consumption. Here, we test specific hypotheses concerning the influence of the observable audience response of laughter, leadership traits, and also perceived charisma. Reagan’s ability to elicit audience laughter sets up following hypothesis that are addressed in two studies:

H1: Laughter in response to Reagan’s humorous comments will increase perceptions of his competence, warmth, and charisma.

Furthermore, due to Ronald Reagan’s effective and prolific use of a range of humour types with strategic intent, we can further test the effect of successful and unsuccessful humour, as marked by the presence of absence of laughter. Specifically, the literature reviewed suggests differential impact of Reagan’s use of self-deprecatory and ridicule humour.

H2: Laughter in response to Reagan’s ridicule of audience members will increase perceptions of his competence.
H3: Laughter in response to Reagan’s self-deprecatory comments will increase perceptions of his warmth.
H4: Laughter in response to Reagan’s humour will increase perceptions of his charisma.

The perception of audience laughter to Reagan’s humour will increase judgments of his leadership competence and approachability. However, this will be dependent on whether the humour is self-depreciatory or directed to other parties. Thus, there will be a main effect of humour on judgments of leadership traits and an interaction between the different types of humour that Reagan displays.

## Methods

### Content coding of the Reagan-Stahl News Story (1984)

The key news story was shown on Thursday, October 4, 1984 via a CBS network primetime television news broadcast, one month before Reagan’s landslide election victory. The news story as analysed had a video clip length of five minutes and forty-five seconds (5:44.85/100s; 345 seconds) with the story length after the introduction by Dan Rather being five minutes and twelve seconds. In the five-minute (306 seconds) news story, Leslie Stahl narrated for just over three minutes (194 seconds), while Reagan had twenty-seven seconds of speaking time dispersed throughout five sound bites. These sound bites all took place during the second half of the news story.

Throughout the news story, two minutes and five seconds of audience applause cheering, laughter, and mixed response could be heard. Applause-cheering can be heard throughout almost two minutes of the story (111 seconds). This is notable because support from partisans in the form of audible responses took place in over one-third of a purportedly critical news-story. While Stahl talked over much of the applause-cheering and mixed applause-cheering and booing, laughter was presented without interruption. Indeed, of Reagan’s five sound bites, three were presented with elicited laughter uninterrupted. The first of these laughter events started at two minutes and forty-five seconds into Stahl’s story, whereas the last occurred just under 2 minutes (114 s) from the end. This news story contained a range of examples of Reagan’s performative style and is thus an ideal means to study the effects of the different types of humour used and the interaction between observable audience responses.

While the placement of the humorous comments did not give Reagan the first or final word in the story, these three laughter-eliciting comments provided him with punctuated support from the audience when he did talk. ANVIL content coding software was used to characterize and analyse the news story [86]. ANVIL allows for frame-by-frame analysis of speaking time and the ability to disambiguate the observable audience responses by considering both audible response by the audience [59], and camera shots of the audience [85]. Adobe Premier Pro software was then used to edit the video and develop the various experimental conditions.

A content analytic approach was applied to the visual coding of the key news report [85]. Specifically, the presence of large (16 of 59 camera shots; 80s and 26.2% of camera time) and approving audiences (15/59 shots; 62.12s and 20.3% of camera time) were coded. When the audible response by the audience is considered in tandem with these types of camera shots, it is found that large, yet non-responsive, audiences were presented in three shots (19.72s), whereas thirteen shots and just over a minute of applause-cheering (60.28s) was heard from large audiences. Audiences seen as approving were evident in fourteen shots for just under one minute (55.52s) where applause-cheering occurred, while laughter was seen in one nearly seven second shot (6.60s). It was expected that the applause-cheering would be most likely observed in media coverage of group settings such as political speeches [75, 76] and intra-party debates [87]. This is due to such observable audience responses predominating in political discourse because of the ease with which candidates can evoke it among supporters in partisan settings. As a result, applause-cheering plays a role as an important barometer of a politicians’ individual appeal during speeches [76, 88] or when in direct competition with other candidates during debates [59]. However, the production decision to incorporate applause-cheering as a major part of a critical news story may be seen as at odds with the perceived intent. So too was the decision to incorporate laughter in response to humorous comments by the then presidential candidate Ronald Reagan.

## Study 1

The objective of the first study is to examine the effects of the observable audience response of laughter and how it moderated the perception of Reagan as an effective presidential leader. It can be expected from the literature reviewed that audience laughter in response to Reagan’s humorous comments will affect perceptions of the leadership traits he holds, whether warmth or competence. The question is, to what extent will the presence or absence of laughter, indicating success or failure of Reagan’s humorous comments differentially affect perceptions of these traits.

While Stahl spoke over the great majority of applause, the first three of Reagan’s sound bites led to observable audience laughter in response to his quips. These occurrences were not spoken over and ensued during a middle portion of the story where Stahl commented on Reagan by stating, “This tight control has baffled those who think that Mr. Reagan is at his very best when he is spontaneous….” With this in mind, three edits totalling just under six seconds (5.^46/100s^ = 2.^93^+.^83^+1.^7^) were made. The video was presented in a between subjects design with three different conditions. The first, presented unedited video as the control condition, with participants seeing what viewers of the CBS news story viewed in 1984. The second two treatments involved either the audience laughter being removed completely, with no noise from the video during the edits, or the audience laughter being faded-out to a level at just under fifty percent of that presented during the original news story. As a result, the treatment effect being considered equals 0.016% of audio-visual time (5.46s/344.83) for the total video.

The first edit took place at 3:19:02 (until 3:21:25) of the video clip after Reagan was shown commenting “I’ll raise his taxes” in response to audience members shown as heckling him. The audience, presumably at a campaign speech held during the Missouri State Fair (based upon the scene prior) responded with loud laughter followed by mixed cheering and applause. At the same time Reagan, shown with his suit jacket off in front of hay bales, displayed a smile of amusement after delivering his punchline and during the audience’s laughter and applause-cheering.

The second edit, of less than a second (3:26:29–3:27:21), took place after Stahl commented positively on Reagan’s ability for “tossing off one-liners,” Here Reagan, dressed in a suit and tie and presumably sitting down for an interview, quipped “I never get good reviews from TASS” after shaking his head, presumably to a difficult question. As a small group of individuals laughed at his response Reagan smiled in amusement.

The third edit (3:41:26–3:43:03) was set up by Stahl as Reagan being “masterful at deflecting a hostile question” when he responded to a reporter at a press conference commenting on his keeping Republican Party representatives in line. Here Reagan responded with a self-deprecatory comment, “How can you say that about a sweet fellow like me?” and laughed while displaying a smile of amusement.

## Participants

Participants were recruited from introductory-level political science classes and were provided extra course credit for taking part in the study. Written consent to participate was obtained prior to taking part. A total of 317 participants took part in the study that lasted from March 2 to April 28, 2018. So as to ensure task compliance, those individuals who stopped engaging within the first 7 minutes and who did not respond to the open-ended prompt “(P)lease list some of the thoughts you had while watching the video clip” were removed from subsequent analysis, which resulted in a final sample of 283 participants. All procedures were approved by the University of Arkansas IRB.

Of these participants, 61.8% identified as female, 81.3% identified as Caucasian (with 5.3% African-American, 2.1% Asian, 8.5% Hispanic, .4% Native American, and 2.5% other ethnicity), and the average age was twenty-one years old (range 18–71, SD = 4.55). The majority of participants identified themselves as identifying with the Republican Party (40.3%), followed by Democratic Party identifiers (35.3%), as independent (15.2%), Libertarian Party (6.7%), Green Party (.7%) and other (1.8%). Random assignment of participants to the different treatments was balanced (unedited video/laughter-in/control [*n* = 96], laughter faded out [*n* = 95], and laughter removed [*n* = 92]) across the three conditions. When tested for randomness in assignment to the treatment condition, we found no statistical bias (all p-values = ns) for sex, ethnicity, age, party identification, and political ideology (social, economic, overall conservative-liberal).

## Procedure

Prior to the taking part in the protocol, participants were asked to provide basic demographic information (age, sex, ethnicity), whether they were registered to vote, the political party they best identify with and their attitudes towards the main US political parties, as well as their own political ideology. At this point, participants were randomly assigned to one of the three different treatment categories (i.e., control condition, laughter faded out or no laughter).

Immediately after the video clips were viewed, participants were first asked to describe their thoughts on the video, how strongly they felt in reference to different emotions at that moment (anxious, proud, angry, reassured, fearful, irritated, disgusted, sad, and happy) on a 0–10 (not at all to extremely). They were then asked their evaluation of the reporter, Leslie Stahl, in terms of their overall impressions of her, as well as how credible, appropriate, and likable she was on a seven-point scale. These items were then combined into an additive index (Cronbach’s a = .873). A final measure, that of how aggressive Stahl was perceived to be, due to weak correlations with the other measures, was analysed separately.

Participants were then asked to evaluate Ronald Reagan’s leadership traits in terms of his *competence*, which was based upon measures of how sincere, aggressive, strong, active, competent he appeared to be (Cronbach’s a = .779); additional measures considered a scale of his *warmth* with questions regarding how intelligent, caring, trustworthy, agreeable, and warm (Cronbach’s a = .928) he appeared during the news story. Responses regarding evaluation of both Leslie Stahl and Ronald Reagan ranged from “Not at all” to “Extremely” on a seven-point (0–6) scale. Finally, to evaluate whether participants noticed the treatment, we asked “How believable did you find the video clip to be?” on the same seven-point scale. Throughout the reported statistical tests an alpha level of >0.05 is designated as n/s.

## Results

Emotional response to the video showed that, how anxious (*F* = .283, *p* = ns), proud (*F* = .465, *p* = ns), angry (*F* = 1.448, *p* = ns), reassured (*F* = .644, *p* = ns), fearful (*F* = 1.848, *p* = ns), disgusted (*F* = .632, *p* = ns), sad (*F* = .192, *p* = ns), and happy (*F* = .119, *p* = ns) participants felt was unaffected by the laughter. However, when least significant differences are considered, participants felt significantly less irritated (*F* = 4.124, *p* = .017, partial η^2^ = .029) when watching the original video (*M* = 3.646) when compared with the treatment videos with laughter faded out (*M* = 2.611, *p* = .008) and laughter completely removed (*M* = 2.793, *p* = .029).

Participant ratings of Leslie Stahl in a similar manner suggested the treatment had little effect. Specifically, the index considering overall performance, perceived credibility, appropriateness, and likability, exhibited no significant violations of homogeneity (*F* [2, 280] = 1.298, *p* = ns) according to the Levene’s test. Analysis of the index shows participants were largely unaffected by whether there was laughter present, faded, or removed entirely (*F* [2, 280] = 0.480, *p* = ns). Likewise, Stahl’s perceived aggressiveness failed to reach statistical significance (*F* [2, 280] = 2.722, *p* = ns).

Similarly, participants did not seem to notice a difference between the different videos. When asked “how believable did you find the video clip to be,” there was no significant difference between the treatments (*F* = 1.005, *p* = ns). In combination with the preceding findings, there was not apparently a cognitively perceived effect from the video as participants were not aware of the treatment.

Analysis of the effect of laughter on evaluation of Ronald Reagan’s leadership traits tells a more nuanced story. Tests for homogeneity of variance regarding the *competence* index finds no significant violations (*F* [2, 280] = 1.536, *p* = ns) as does the between-subjects ANOVA between the three groups: *F* [2, 280] = 2.677, *p* = ns. Although the patterns of response mirror those of perceived *warmth* (Laughter in M = 23.80; Laughter faded out M = 22.31; Laughter removed M = 22.20).

When Levene’s test for homogeneity of variance regarding the index of Reagan’s *warmth* is considered, no significant violations were found (*F* [2, 280] = 1.699, *p* = ns). However a highly significant between-subjects effect across the three humour groups was revealed: *F* [2, 280] = 4.078, *p* = .018, partial η^2^ = .028. Here, Reagan’s trait evaluations were enhanced by the presence of the loud laughter evident in the original news story, with post-hoc comparisons showing that the laughter remaining condition (M = 25.94, p = 0.05) was significantly greater than the faded-out condition (M = 23.39) and the complete removal of the laughter (M = 23.60). Thus, while perceptions of his *warmth* are all relatively high, they are significantly reduced by the laughter either being faded out or removed entirely (p = ns).

Finally, as a control item a single measure of how humorous participants thought Reagan to be was included. No significant violations of homogeneity were found (*F* [2, 280] = 1.497, *p* = ns) and the pattern revealed was similar to that regarding Reagan’s *warmth* index. Namely, a significant between-subjects effect between the three humor groups, *F* [2, 280] = 3.411, *p* = .034, partial η^2^ = .024. When post-hoc least significant differences are considered, there were no significant differences between the laughter faded out (M = 5.13) and laughter completely removed (M = 4.96) groups, as was the case with perceptions of Reagan’s *warmth*. However, Reagan was considered significantly more humorous with the laughter in (M = 5.52) at the .05-level.

## Discussion

The finding that observers’ emotion was largely unaffected by the treatment, with the exception of feeling irritated, is perhaps not unexpected. The treatment, which is comprised of less than five seconds of laughter across the three Reagan excerpts, is a subtle and unobtrusive stimulus that potentially would not have an observable effect on self-reports of introspective evaluation of emotional response. Furthermore, by only considering between-subjects effects we cannot tease out whether the news story had a greater influence on participant emotional response and how this might have differed across the treatments. Even though the sole finding concerning irritation was aligned too the expectations both in the pattern of response, with greater irritation felt by those either not hearing laughter or diminished laughter, suggesting a failed humour attempt, and the comparatively weak effect of the treatment, future studies should consider change in self-reported emotional state through within-subjects design.

The lack of significant effect on evaluation of the reporter, Leslie Stahl, is likely due to the average age of the participants, which may have rendered her work as a nationally known figure unknown. While Ronald Reagan is recognized as a Republican Party icon and is mentioned in both glowing and critical terms by participants, Stahl is not so well recognized. As noted by Vraga and colleagues [53] when comparing participant response to a famous U.S. talk show host and an unfamiliar moderator “… a laugh track has very different effects when a host is a well-known comedian versus an unknown talk show host." (p.143) In other words, the perceptions of newscaster Stahl and the presentation of Reagan’s (un)successful humour may be premised upon the humour being interpreted as a benign violation of expectations [89], as opposed to ridicule that is received as more aggressive and less socially acceptable.

## Study 2

Type of humour likely play a role in perceptions of Ronald Reagan and how he is portrayed in this news story. As noted by Baumgartner, not only does “prior knowledge of the target of the humour affects susceptibility to attitude change” but also the context of political humour plays a role [90]. Whether the humour is other-deprecatory and ridicule-oriented or self-deprecatory plays a role in its perceptions especially upon considering the audience [60] when Reagan ridicules an audience member. Because of Reagan’s standing as a Republican Party icon, the effect of the audience’s response to his rejoinder to the dissenter within the audience might be accentuated if participants perceive his response being received in a less than flattering manner. This finding is consistent with considerable prior research considering the target of the humour, especially political figures [60, 90–94].

The first experimental study is extended here by including control and full treatment levels, with all three laughter events present or removed; this allows replication of the first hypotheses regarding responses to laughter in the evaluation of leadership competence and warmth. This study will also examine the presence of laughter in response to Ronald Reagan’s humour and the effect that it will have on his perceived charismatic traits. The influence of specific laughter-eliciting comments removing concomitant laughter to consider the influence of different types of (un)successful humour will also be examined here. As a result, the second experimental study will have five different levels.

Additionally, the charisma of presidents is driven in part by perceived leadership traits of *competence* and *warmth* [68]. Even with participants not knowledgeable about Reagan, the positive visuals as well as the extensive applause-cheering throughout the news report, whether included inadvertently or not, does convey his charismatic presence. However, whether *charisma* plays a moderating or mediating role in conjunction with the observable audience response of laughter is still in question.

## Method

The second study utilizes three edits that totalled just under six seconds (5.46/100s = 2.93+.83+1.7) with a five condition between-subjects design. The first condition presented unedited video as a control with participants seeing what the 1984 CBS news viewers saw. The second replication treatment removed all three observable audience responses of laughter completely, with no noise from the video during the edits leading to the treatment effect 5.46 seconds of the total video (344.83s).

The next three conditions involved the removal of laughter from the three specific humorous comments. The third treatment, taking place from 3:19:02 until 3:21:25 of the video, showed Reagan responding to a heckler with the comment “I’ll raise his taxes” eliciting loud laughter followed by mixed cheering and applause. The fourth treatment involved the removal of less than a second of laughter from a small group of individuals and occurred from 3:26:29–3:27:21 of the video when a seated Reagan quipped “I never get good reviews from (the Russian news agency) TASS” after shaking his head. The final treatment condition saw Reagan use self-deprecatory humour to deflect an aggressive journalist’s question, leading to brief laughter at 3:41:26–3:43:03 of the video.

## Participants

A power analysis using G*Power was carried out to determine sample size. Here, the traditional power estimation parameters for the least explained variable, the trait of *competence*, (1 –β err probability = 80%; α error probability = .05; effect size of f = .136). Findings based upon the effect likely, given the means and standard deviations uncovered in experimental study one, suggests a sample size of 650 participants would be required.

Participants were recruited using a snowball sampling approach in which upper-division undergraduate students received course credit for taking part in and recruiting participants. To better reflect the general population, older participants were systematically recruited, leading to a more age diverse sample. A total of 1041 individuals entered the study that lasted from November 16, 2020 to November 11, 2021; of those 315 did not spend at least seven minutes (420 seconds) in the study and were removed as per the previous study parameters. An additional 60 participants were removed due to their not responding to the open-ended prompt and a further 15 for not answering any post-treatment questions, leaving a total of 651 participants in the study. All ethical considerations, including consenting of the participants were identical to that reported for study 1.

Of those taking part, 61.4% identified as female, 83.3% identified as Caucasian (with 3.7% African-American, 0.5% Asian, 7.5% Hispanic, 2.6% Native American, and 2.3% other ethnicity); the average year of birth was 1982 old (range 1934–2005, SD = 16.3). The majority of participants identified themselves as identifying with the Democratic Party (38.9%), followed by Republican Party identifiers (33.3%), as independent (16.7%), Libertarian Party (3.9%), Green Party (1.4%) and other (5.6%). Random assignment of participants to the different treatments was balanced. We first replicated study 1 by having the unedited video control condition [*n* = 119] and the treatment condition with all laughter removed [*n* = 139]. The other three conditions considered the effect of removing individual laughter events, with the first removing laughter from Reagan’s response to a heckler [*n* = 126], the second removing small group laughter [*n* = 131], and the third removing group laughter in Reagan’s response to journalistic aggression [*n* = 136]. When tested for randomness in assignment to across the five treatment conditions, we found no statistical bias (p = ns in all cases) for sex, ethnicity, age, party identification, and political ideology (social, economic, overall conservative-liberal).

## Procedure

As was the case with the first experimental study, participants were asked basic demographic questions (age, sex, ethnicity), as well as questions about whether they were registered to vote, the political party they identify with and self-reported political ideology. Additionally, they were asked to state how familiar they were with President Ronald Reagan, especially as this more externally valid sample had a greater distribution of ages and experience with Reagan, potentially influencing response. The distribution of participants was therefore examined as a separate, exploratory, and hypothesis-generating model with this variable as a moderator.

However, as the first experiment suggested differences in response to the video treatments, participants were asked to state their feelings both prior to and immediately after the presentation of the stimuli in terms of their emotions at that moment (anxious, proud, angry, reassured, fearful, irritated, disgusted, sad, and happy) on a 0–10 (not at all to extremely) scale. The evaluation of perceived *charisma* was based upon whether “This leader…” “moves people toward a goal,” “has a vision,” “inspire dares to take risks,” and “elicits a feeling of involvement in me.” [69]. The resulting scale showed strong reliability (Cronbach’s a = .865).

In line with the first experimental study, participants were asked to evaluate the reporter, Leslie Stahl, based upon their overall impressions of her, as well as how credible, appropriate, and likable she appeared in this video (Cronbach’s a = .919). Participants were also asked to evaluate Ronald Reagan’s leadership traits in terms of his *competence*, based upon measures of how sincere, aggressive, strong, active, competent he appeared to be (Cronbach’s a = .826). We also consider perceptions of his *warmth* with questions regarding how intelligent, caring, trustworthy, agreeable, and warm he appeared to be during the news story (Cronbach’s a = .908). All these were measured on a seven-point (0–6) scale ranging from “Not at all” to “Extremely”.

## Results

Change in emotional response from immediately before watching the video to immediately afterwards using repeated-measures ANOVA suggests that the video had a significant effect on how participants felt across all emotions (see Table 1). There was a small effect with a slight increase in fear (pre M = 1.567, se = .092; post M = 1.775, se = .097); sadness likewise showed a slight increase (pre M = 1.600, se = .088; post M = 2.059, se = .101) with a small-to-medium effect size, whereas felt anxiety decreased (pre M = 3.177, se = .115; post M = 2.719, se = .112) to a small-to-medium extent due to the video.

**Table 1:** Results from the various repeated measures ANOVA for each affect tested in study 2 (F-test, significance, partial η^2^).

	Change	Change*Treatment
Anxiety	*F* = 68.169 *p* < .001 **η**_p_^2^ = .042	*F* = 1.476 *p* = .208 **η**_p_^2^ = .009
Fear	*F* = 6.330 *p* = .012 **η**_p_^2^ = .010	*F* = 2.152 *p* = .073 **η**_p_^2^ = .013
Anger	*F* = 130.061 *p <* .001 **η**_p_^2^ = .168	*F* = 3.704 *p* = .005 **η**_p_^2^ = .022
Irritated	*F* = 125.913 *p <* .001 **η**_p_^2^ = .163	*F* = 1.003 *p* = .405 **η**_p_^2^ = .006
Disgusted	*F* = 210.919 *p* < .001 **η**_p_^2^ = .246	*F* = 1.132 *p* = .340 **η**_p_^2^ = .007
Sadness	*F* = 26.649 *p* < .001 **η**_p_^2^ = .040	*F* = .609 *p* = .656 **η**_p_^2^ = .004
Reassured	*F* = 120.481 *p* < .001 **η**_p_^2^ = .157	*F* = .619 *p* = .649 **η**_p_^2^ = .004
Proud	*F* = 79.406 *p* < .001 **η**_p_^2^ = .109	*F* = .990 *p* = .412 **η**_p_^2^ = .006
Happy	*F* = 264.330 *p* < .001 **η**_p_^2^ = .290	*F* = .484 *p* = .747 **η**_p_^2^ = .003

Emotions associated with pleasantness and positive appraisal all decreased as a result of the video, showing either medium-to-large (proud pre-M = 4.002, se = .129) or large (reassured pre-M = 3.412, se = .122; post-M = 2.174, se = .105; happy pre-M = 5.843, se = .108; post-M = 4.272, se = .122). For their part, the negative appraisal emotions of irritated (pre-M = 1.786, se = .093; post-M = 3.101, se = .114), disgusted (pre-M = 1.023, se = 077; post-M = 2.540, se = .116) and anger (pre-M = 1.070, se = .076; post-M = 2.173, se = .116) all increase with the video having a large effect size.

While all emotional state measures changed because of the video, only anger was affected by the treatment condition. As can be expected, the least amount of increased anger came in the treatment with all three laughter elements present; while the four other treatments between the video with laughter and with it removed failed to reach statistical significance.(M = 1.626, se = .172, p =.ns; vs M = 1.567, se = .170, *p* = ns), significant differences only occurred when anger in the laughter-present video (M = 1.223, se = .181) was compared with all laughter absent (M = 1.745, se = 167, *p* = .034) and with the first treatment condition in which the first laughter utterance was removed (M = 1.948, se = .167, *p* = .004).

Participant ratings of Leslie Stahl in a similar manner suggested the treatment had little effect. Specifically, the index considering overall performance, perceived credibility, appropriateness, and likability, exhibited no significant violations of homogeneity (*F* [4, 686] = 1.070, *p* = ns) according to the Levene’s test. Analysis of the index shows participants were largely unaffected by whether there was laughter present, faded, or removed entirely (*F* [4, 686] = 0.387, *p* = ns). Likewise, Stahl’s perceived aggressiveness (*F* [2, 280] = .174, *p* = ns) failed to reach statistical significance.

Similarly, participants did not seem to notice a difference between the different videos. When asked “how believable did you find the video clip to be,” there was no significant difference between the treatments (*F* = .242, *p* = ns). In combination with the preceding findings, there was not apparently a cognitively perceived effect from the video as participants were not aware of the treatment.

Analysis of the effect of laughter and its removal from the video treatment on evaluation of Ronald Reagan’s leadership traits does not replicate the first experiment. Tests for homogeneity of variance regarding the *competence* index finds no significant violations (*F* [4, 686] = 1.682, *p* = ns). The between-subjects ANOVA between the five groups does not reveal significant differences: *F* [4, 686] = 1.313, *p* = ns.

Levene’s test for homogeneity of variance revealed a significant violation (*F* [4, 686] = 2.480, *p* = .043) when considering the index of Reagan’s *warmth*. When the Brown-Forsythe robust test was used, no significant between-subjects effects across the five humour groups was revealed: *F* [4, 686] = 1.299, *p* = ns. Likewise, while with perceptions of Reagan’s charisma no significant violations of homogeneity of variance were found using Levene’s test (*F* [4, 686] = 1.919, *p* = ns); no significant between-subjects effects across the five humour groups was revealed; *F* [4, 686] = .516, *p* = ns.

Finally, as a control item a single measure of how humorous participants thought Reagan to be was included. Significant violations of homogeneity were found with the Levene test (*F* [4, 686] = 5.377, *p* < .001), yet no significant differences between the groups were seen, *F* [4, 686] = .589, *p* = ns, when the Brown-Forsythe robust test was used.

## Discussion

The second study provides insight regarding the importance of the population used and methods employed. First, by using a more representative sample in terms of age, with the first study’s average age being twenty-one years old, and the second study’s average age of thirty-nine years old, we can expect that perceptions of President Ronald Reagan, to be well-established for good or bad. While a historical figure, allowing us to carry out an experiment over a long period of time without worries over external validity, Reagan remains a powerful political symbol in terms of social identity. Indeed, when considering the distributions on the constructs of charisma and warmth, eight percent of participants held a ceiling perception of him on both measures. Thus, even though age, gender, and party identity were randomly distributed through the different treatments, the likelihood of such a weak treatment—between less than a second of laughter to six seconds of laughter—embedded within a roughly five-minute video having an effect was diminished.

Second, the use of trait measures may not be sensitive enough to capture contemporaneous stimuli, especially regarding well known figures (and even those not so well known as in the case of Leslie Stahl). That we found significant and predictable change in all the participant emotional state self-report measures prior to and after watching the video, and that anger was most affected by the absence of laughter, both overall and in Reagan’s response to the heckler, suggests that the presence of laughter does have an effect on participants–even ones with strongly held opinions.

## General discussion

Our findings cohere with the expectations of Vraga and colleagues [53] that when people have limited information to deal with ambiguous situations, they will rely upon subtle signals–especially those socially influential and reliable indicators of positive regard as audience laughter. In this paper, we find two substantially different groups of study participants responding in line with Vraga and colleagues’ results, as the much younger–and likely less politically knowledgeable–study 1 participants used audience laughter, or its absence, as a factor in their evaluating Ronald Reagan’s warmth and, to a lesser extent, competence. The older and more politically experience and involved experimental study 2 participants were not affected by audience laughter’s presence or absence in their evaluation of Reagan’s leadership traits. This was likely due to either experiencing Reagan as an active and polarizing political figure or as seeing him as a historically relevant political figure.

The second, subtle, and perhaps more compelling indicator that audience laughter does have an effect on participants lies with the indicators of appraised emotion. In the first experiment, there were between-subject treatment differences in felt irritation, with participants feeling less irritated when viewing the video with the laughter in than with the video with the laughter removed or faded out. While experimental study 2 participants felt irritation was not significantly affected, their felt anger was. In other words, the older and more politically experienced participants had a response in the same emotion family that replicated that of irritation, with those not hearing audience laughter more angry than those who did hear audience laughter, and both studies having similar effect sizes. Furthermore, the experimental extension in the second study, which teased out the effects of the success–as measured by audience laughter or its absence–of humorous statements found that Reagan’s aggressive quip in response to protesters (“I’ll raise his taxes”) had the strongest treatment effect when post-hoc comparisons were made, stronger even than all laughter removed. This suggests, in line with Stewart’s [60] finding that other-deprecating and aggressive humour, including ridicule, can be dangerous for a leader if supporters are not there to respond to a quip or joke with laughter.

Taken together, these findings point to a greater awareness of how even very subtle stimuli might affect various measures differently, especially given distinct populations. Having multiple measures thus not only makes sense in assessing discriminant validity of treatment effects it also provides for greater comprehension of how individual differences exhibit themselves. Because the traits of warmth, competence, and charisma can be seen as the crystallization of emotional appraisals in response to individuals over a period of time—albeit one that is more malleable in the absence of pre-existing information–choosing and paying attention to distinct measures based upon population characteristics makes eminent sense when planning a study. It also points towards the more extensive use of highly responsive measures of affect, such as provided by psychophysiology, when crafting an experiment and viewing appraisal and response as a continuum affected by multiple internal and external factors.

## Conclusions

Perhaps the most pertinent finding from this paper pertains to the use of an externally valid stimulus that, while nearly forty years old, still resonates today both in experimental effects and lessons imparted. First, historically relevant stimuli remain impactful, as can be seen by the cornerstone work by Fein, Goethals, and Kugler [9] upon which this paper builds, as the fresh eyes (and brains) of undergraduates in our first experiment had their perceptions significantly affected nearly three decades after Ronald Reagan left the presidency. Perhaps more important is that such a minor treatment in our study–up to 5 ¾ seconds removed from a five-minute+ video–had a small-to-moderate effect size suggests that even perceived minor production choices can have subtle, yet impactful implications for the perceptions and choices of low-information voters reliant on the social influence of others. Despite the fact that the key news story was produced decades ago the use of humour is often seen in contemporary political settings. Future work exploring the social psychological effects of different types of humour that is displayed by politicians should focus on the interactions between humour types and the strength of the observable audience response. As we have shown here it is the interaction between the two that impacts audience perceptions, in turn likely shaping attitudes and, potentially, behaviour.
